# The Camerino symposium series (1978–2013): a privileged observatory of receptorology development

**DOI:** 10.1186/2193-9616-1-21

**Published:** 2013-12-20

**Authors:** Mario Giannella, Piero Angeli

**Affiliations:** School of Pharmacy, Medicinal Chemistry Unit, University of Camerino, 62032 Camerino, Italy

**Keywords:** G Protein-Coupled Receptor (GPCR), Camerino symposium, Receptorology, Mutagenesis, Chimeric receptor, Orphan receptor

## Abstract

The organizers of the Camerino Receptor Symposia survey the development of receptorology. They trace the course from the first Symposium in 1978, which laid the foundation for Pirenzepine, the first selective muscarinic antagonist, to the 2010 Symposium, which highlighted the utility of functional simple domain antibodies (nanobodies) as novel G Protein-Coupled Receptor (GPCR) modulators. This 30-year period sees the acceptance of terms such as G-protein, auto- and heteroreceptors, site-directed mutagenesis, chimeric receptors, constitutive activity, inverse agonism, and orphan receptors. GPCRs are finally a reality and Langley and Ehrlich, if they returned to their laboratories, would be proud of how their intuitions have been realized.

## Review

"G protein-coupled receptors are integral membrane glycoproteins, containing a seven-transmembrane helical protein-fold, that mediate a variety of signaling processes, such as neurotransmission, hormonal response, olfaction, and light transduction." It’s been a long journey to arrive at this definition. To us, the path really began on Monday September 11, 1978, at Camerino, when the old room of the Chemistry Institute filled with researchers interested in studying receptor chemistry that was still a very young subject. Despite the publication of several physiological and biochemical works, the physical existence of receptors remained controversial. This skepticism was expressed by Raymond Ahlquist, a respected pharmacologist. Even though he had differentiated the adrenoreceptors as α and β in 1945, Ahlquist still wrote in 1973 "*This would be true if I were so presumptuous as to believe that α and β receptors really did exist. There are those that think so and even propose to describe their intimate structure. To me they are an abstract concept conceived to explain observed responses of tissues produced by chemicals of various structure*" (Ahlquist 1973).

Sitting in the front row at the Chemistry Institute that day were Bernard Belleau, Philip S. Portoghese, Peter G. Waser, and Pietro Pratesi, the leader of one of the few Italian teams devoted to studying receptors, particularly the correlation between the chemical-physical properties of sympathomimetic amines and their biological activity (Pratesi 1958). These four researchers had been invited to Camerino as Speakers at the International Symposium on 'Recent Advances in Receptor Chemistry’ by our research team, whose reputation was based on just one paper published (after careful revision!) in the *Journal of Medicinal Chemistry* (Gualtieri et al. 1974).

During the four-day meeting, receptor theory, neurotransmitter membrane receptors, quantitative structure-activity relationships, and computer procedures for rationalizing drug-receptor interactions were the subjects of lively discussion and debate, particularly energized by E. J. Ariëns who, in 1965, had established the prestigious monograph series *Molecular Pharmacology,* published by Academic Press (Ariens 1965). In his opening lecture, Ariëns hypothesized the existence of sites of action and sites of binding and therewith the existence of silent receptors in membrane proteins with receptor functions. At that time, agonists and competitive antagonists, each of them binding to their own specific receptor sites (two-state model), were thought to be linked by an allosteric interaction. The exceptional scientific atmosphere of the meeting fostered the beginning of a collaboration between Nigel Birdsall, one of the young English talents more involved in receptor study, and Rudolf Hammer, an authoritative researcher at the German pharmaceutical company Boehringer Ingelheim. Birdsall and Hammer eventually worked together to produce an extensive study of Pirenzepine, the first selective muscarinic antagonist, which led to our knowledge of muscarinic receptor heterogeneity. Nevertheless, the content of receptorology then was still so vague that Belleau, in the Preface of the Proceedings published at the end of the meeting, stated "*The hypothetical borders delineating the field of receptorology are so vague and fuzzy that it is hardly possible to provide a clear definition of that science"* (Gualtieri et al. 1979). As an example, transduction mechanisms anticipated that a receptor, interacting with its hormone, could link and activate the enzyme adenylyl cyclase (mobile receptor hypothesis) so forming the second messenger cAMP (Cuatrecasas et al. 1975).

The discovery of a protein acting as transducer between membrane receptor and adenylyl cyclase significantly increased our knowledge of the molecular events that convey signaling from the outside to the inside of the cell. Alfred Gilman, after purifying this protein, called it Gs-protein (Gilman 1987). At the beginning of the 1980s, a number of observations lead to the introduction of the 'ternary complex model’ to describe the receptor interaction between G-proteins and endogenous ligands (De Lean et al. 1980) and the quantitation of high (G-protein coupled) and low (not coupled) affinity states of the receptor (Kent et al. 1980). Several novel technologies were developed, including radioligand binding and affinity labeling techniques, detergent solubilization, affinity chromatography purification, and lipid reconstitution. These enabled the fruitful and effective isolation and characterization of receptor processes. For example, the new binding affinity techniques were applied to new large natural and synthetic compounds. This led to the discovery of receptor subtypes in what was previously thought to be a homogeneous system.

Interdisciplinary collaboration between medicinal chemists, pharmacologists, biochemists, and molecular biologists was essential to achieving these advances. This was recognized by the first Camerino Symposium and by every subsequent edition. Such interdisciplinary collaboration led to the isolation and purification of the β_2_-adrenoreceptor and its characterization as a glycosylated and phosphorylated polypeptide chain of MW ~ 60–65000 Da (Benovic et al. 1984). The next step was the reconstitution in phospholipid vesicles of this protein and the verification of its functionality maintenance (Cerione et al. 1983). These advances were reflected in the 1983 meeting, 'Highlights in Receptor Chemistry’ , whose main topics included Langer’s work in describing presynaptic receptors in 1978 (Langer 1978) (subdivided at Camerino for the first time into auto- and hetero-receptors), the description of the dopaminergic receptor’s topography with the 'receptor mapping’ technique, and a first application of computational procedures in classifying drug and receptor congeners. In the opening article of the Proceedings, David Triggle wrote "*From cloudy and uncertain beginnings we now with confidence can discuss receptor structures, coupling, diseases, defects and can use this knowledge to design new pharmacologic and therapeutic tools*" (Melchiorre and Giannella 1984).

The advent of recombinant DNA technology in the 1980s provided new knowledge of the amino acid sequence of receptors. At the same time, their molecular mechanism of activation was explored using site-directed mutagenesis, chemical synthesis, and molecular modeling in a combined approach. Together with computer graphics, valuable information was obtained concerning a receptor’s three-dimensional structure and the specific amino acids involved in a given interaction. The β_2_-adrenoreceptor was the first to be cloned and its architecture acknowledged as a homologue of the visual pigmet rhodopsine (Dixon et al. 1986), whose entire amino acid sequence had been determined in 1982 (Ovchinnikov 1982). Hypotheses on the functioning mechanism of the receptor revealed a linkage between the receptor sequence and G-protein transduction. For this reason, researchers began to think that most GPCRs might share a similar arrangement (Dohlman et al. 1987). Robert Lefkowitz, who won the Nobel Prize in Chemistry with Brian Kobilka in 2012 for their pioneering work in studying seven transmembrane receptors (7TMRs, ironically called "The magnificent seven" by Lefkovitz) wrote "*I never imagined that the superfamily of 7TM receptors would grow so large and diverse*" (Lefkowitz 2004). Indeed, ions, organic odorants, amines, peptides, proteins, lipids, nucleotides, and even photons were identified as possible agents able to mediate their message through the 7TMRs. In 1987, it was even discovered that some gases could perform a similar role, with nitric oxide (NO) being the first such finding (Palmer et al. 1987). John Vane, Nobel Prize winner in Physiology and Medicine in 1982, took part in the 1987 Camerino Symposium. In his opening lecture 'Adventures in Bioassay’, he wrote of the "*pharmacology and physiology surprise… that one of the most fascinating mediators is a simple one-to-one combination of the main elements of the atmosphere"* (Melchiorre and Giannella 1988).

At the end of the 1980s, Fulvio Gualtieri’s lecture on 1,3-oxathiolane isosteric analogs of muscarinic 1,3-dioxolane ligands (Gualtieri et al. 1988) suggested to Bernard Belleau the synthesis of Lamivudine, a powerful inhibitor of reverse transcriptase (introduced in the following symposium). In this molecule, the 1,3-oxathiolane scaffold, bound to a pyrimidine ring, simulates the ribose nucleus (Soudeyns et al. 1991). This edition of the Symposium saw the beginning of an Italo-Dutch collaboration through a scientific twinning between the Camerino group and a group at Vrije Universiteit (Amsterdam) led by Prof. Henk Timmerman. In the 2007 edition, 'An Overview of Receptor Chemistry’ , this collaboration was extended to the Cyprus Conference held in Limassol and directed by Prof. Alexandros Makriyannis, director of the Center for Drug Discovery at Northeastern University (Boston). SAR studies yielded more and more selective compounds, allowing the differentiation of many receptor subtypes. As per Fisher’s metaphor (Fischer 1894), these were the keys that unlocked the labyrinth. At the same time, the mechanisms preceding and following ligand-receptor interaction were also studied.

The first mutagenesis studies involved the design of structures resulting from the combination of the sequences of multiple receptors (chimeric receptors) (Ostrowski et al. 1992; Strader et al. 1994) or structures with one or more mutated amino acids in specific regions of the receptor polypeptide (site-directed mutagenesis). One of the first chimeras was created by stitching together different sections of α_2a_- and β_2_-adrenergic receptors (Kobilka et al. 1988). It showed that residues in the membrane span produce the ligand-binding specificity, whereas the sequences in the amino and carboxyl terminal portion of the third intracellular loop produce the specificity binding to Gs or Gi. Equally important are the results that Susanna Cotecchia obtained by modifying four amino acids of the third cytoplasmic loop of the α_1B_-adrenergic receptor (Cotecchia et al. 1992). She presented these results at the 1999 symposium (Cotecchia et al. 2000). These approaches elucidated the role of specific regions of the sequence of the polypeptide chain or of single amino acids, some of which gave rise to constitutively activated receptors. The probable elimination of intermolecular interactions, which are essential in keeping the receptor in an inactive conformation, gives rise to signals that are similar to those of the agonists. As a consequence, it was possible to assume that naturally occurring mutations caused various diseases, including some proliferative disorders (Spiegel 1998). These observations also led to the discovery of inverse agonism, which is an opposing phenomenon of the constitutive activity, presumably induced by binding and stabilizing the receptor in the inactive state (Lefkowitz 1993). For this reason, inverse agonists are also useful and effective therapeutic tools.

Another important and still unsolved challenge for researchers is the receptor characterization of unknown ligands or functions named 'orphan receptors’ , obtained with the cloning techniques, whose deorphanization can lead to the discovery of novel physiological responses. The first example of deorphanization was the 5-HT_1A_ receptor encoded by the clone 'G21’ , isolated from a size-selected human genomic DNA library (Fargin et al. 1988). To date, in spite of the many studies by groups all over the world, only 4% of the proposed pharmacologically relevant 7TMRs are known. Some of the strategies devised to identify the natural ligands of orphan GPCRs were one topic of discussion at the 2007 symposium sessions.

At the end of the 1990s, researchers had defined the universal mechanism that regulates receptor function, which is a sequence of stimulus-dependent receptor phosphorylation by the kinase enzymes (GRKs) followed by arrestin binding (Pitcher et al. 1998; Kohout and Lefkowitz 2003). Thus, Triggle remarked in his opening lecture of the 1999 Symposium "*By the beginning of the 20th Century the foundation had been laid for a definition of receptors that embodied the concepts of specificity, including stereoselectivity, dose–response relationships and transduction-concepts still in use today*" (Triggle 2000). At the beginning of the third millennium, it is possible to synthesize receptors, define their character and properties, and produce genetically modified animals that display our own human receptors. The time is now ripe for advancing our knowledge of those complex mechanisms, which have so fascinated researchers through the years that, in his 'Historical Review’ in 2004, Lefkowitz dedicated to them "*entirely his research career*" (Lefkowitz 2004). Receptors can have many faces and acts, as monomeric proteins, as dimers (especially heterodimers), or as oligomers (multimeric quaternary structures). For example, Roberto Maggio’s lecture at the Third Millennium Symposium demonstrated that, when co-expressed in the same cells, the M_2_ and M_3_ muscarinic receptor subtypes can cross-interact with each other forming a chimeric muscarinic M_2_-trunc/M_3_-tail receptor with new pharmacological properties (Chiacchio et al. 2000). Consequently we could improve or change our strategies for drug design and development and drug-receptor interaction. The advent of genomics provided new genetically defined targets, which could be associated with disease states, providing new research tools with which to define and validate targets such as knockout mice, siRNA, and so on. The 2003 Symposium, 'Ongoing Progress in Receptor Chemistry’ , highlighted new tools for medicinal chemists. These included combinatorial chemistry, extremely useful in both generating 'hits’ and exploiting molecular space around a 'lead’ structure, template-guided synthesis or 'click chemistry’. Moreover, in the 2003 symposium, computational techniques for the study of GPCRs and the rational identification of their ligands are introduced, such as bi-dimensional (2D) and three-dimensional quantitative structure-activity relationships (QSAR), pharmacophore searches, and virtual screening (Triggle 2004).

There was increasing therapeutic interest in molecules which could bind one or more allosteric sites and positively or negatively modulate (PAMs or NAMs) the endogenous ligand response, or which themselves had an agonist or antagonist activity (ago- or antago-allosteric modulators) (Keov et al. 2011). This approach can improve the ligand’s subtype selectivity, due to the higher diversity of the allosteric domain relative to the orthosteric one (Christopoulos 2002). Moreover, the allosteric modulators impose a 'ceiling’ on the magnitude of their effect (May et al. 2007). These studies led researchers to coin the term 'cooperativity’ (postive or negative) to indicate the action of molecules which, by interacting with orthosteric or allosteric sites of one of the two receptors that are part of the homo- or heterodimer, alter the same sites’ binding propensity of the other protomer (Milligan and Smith 2007). The introduction of allosteric modulators to the system demands further revision and expansion of the ternary complex model, explaining the drug behavior, that was presented by Nobel price Whyte Black in his opening lecture "The pharmacology of receptors at the physiological level" of 1991 symposium. Specifically, the model evolved to the 16-point quaternary complex model. This model takes into account the concomitant binding of orthosteric and allosteric ligands and G protein on the receptor, which can exist in active and inactive conformational states (Christopoulos and Kenakin 2002; Bridges and Lindsley 2008). The selectivity can be engendered by combining both ortho- and allosteric pharmacophores within the same molecule to yield a novel class of 'bitopic’ or 'dualsteric’ GPCR ligands. This multitarget approach, which somewhat overthrows the one-molecule-one-target paradigm, has been widely applied in the treatment of neurodegenerative and tumor diseases, where a variety of pathological disorders is indicated. Due to the novelty and potential of this therapeutic strategy, an entire session was devoted to the topic at the 2010 Symposium 'Trekking Through Receptor Chemistry’. At the 2010 Symposium, the utility of functional simple domain antibodies (nanobodies) as novel GPCR modulators was illustrated.

Throughout the years, the symposia saw an increasing emphasis on computational techniques. In particular, as we discussed, talks on computer-aided drug discovery became a central part of the Camerino meetings since the early 2000s and underwent a rapid expansion hand in hand with the flourishing of GPCR structural studies. The solution of the crystal structure of rhodopsin in 2000 provided for the first time a reference three-dimensional model (template) for the whole family A of GPCRs (Palczewski et al. 2000). For the following seven years, rhodopsin remained the only available receptor solved crystallographically. However, starting in 2007, the field of GPCR structural studies experienced a dramatic expansion. At the moment twenty-one are the GPCRs for which medium to high resolution crystal structures have been solved, in most cases solved in complex with multiple small molecule ligands (agonists or antagonists) (Congreve et al. 2011). Among the various structures of GPCRs that were recently solved crystallographically are those of the M_2_ (Haga et al. 2012) and M_3_ muscarinic receptors (Kruse et al. 2012), which have been for many years one of the main foci of our research and the symposium.

## Conclusion

Let this issue’s authors describe the latest developments for the pleasure of the readers since we were lucky enough to hear them personally at our last Symposium during the "G Protein-Coupled Receptors: Finally a Reality" session. As a closing comment, we would quote an evocative line from David Triggle (Triggle 2000): "*Langley and Ehrlich might today be strangers in a strange land were they to return, but they would surely recognize the magnificent fruits of their toil in the vineyards*".

## Authors’ information

Mario Giannella is Emeritus Professor of Medicinal Chemistry at Camerino University. Piero Angeli is Full Professor in Medicinal Chemistry and Coordinator of the 'Pharmaceutical Sciences’ PhD at Camerino University’s School of Advanced Studies.

## Abbreviations

GPCR: G protein-coupled receptors
QSAR: Quantitative structure-activity relationships
PAMs: Positively allosteric modulators
NAMs: Negatively allosteric modulators
7TMRs: Seven transmembrane receptors.
